# Estimation of renal function in the intensive care unit: the covert concepts brought to light

**DOI:** 10.1186/2052-0492-2-31

**Published:** 2014-05-07

**Authors:** Sham Sunder, Rajesh Jayaraman, Himanshu Sekhar Mahapatra, Satyanand Sathi, Venkata Ramanan, Prabhu Kanchi, Anurag Gupta, Sunil Kumar Daksh, Pranit Ram

**Affiliations:** Department of Nephrology, Post Graduate Institute of Medical Education and Research (PGIMER), Dr. Ram Manohar Lohia Hospital, 15/57, Second floor, Old Rajinder Nagar, New Delhi, Delhi, 110060 India

**Keywords:** Renal function in ICU, Augmented renal clearance, Cystatin C, MDRD, Optic ratiometric method for GFR

## Abstract

Frantic efforts have been made up to this date to derive consensus for estimating renal function in critically ill patients, only to open the Pandora's box. This article tries to explore the various methods available to date, the newer concepts, and the uncared issues that may still prove to be useful in estimating renal function in intensive care unit patients. The concept of augmented renal clearance, which is frequently encountered in critically ill patients, should always be taken into account, as correct therapeutic dosage of drugs sounds vital which in turn depends on correctly calculated glomerular filtration rate. Serum creatinine and creatinine-based formulae have their own demerits that are well known and established. While Cockcroft-Gault and 4-variable modification of diet in renal diseases formulae are highly inadequate in the intensive care setup for estimating glomerular filtration rate, employing isotopic methods is impractical and cumbersome. The 6-variable modification of diet in renal diseases formula fairs better as it takes into account the serum albumin and blood urea nitrogen, too. Jelliffe's and modified Jelliffe's equations take into account the rate of creatinine production and volume of distribution which in turn fluctuates heavily in a critically ill patient. Twenty-four-hour and timed creatinine clearances offer values close to reality although not accurate and cannot provide immediate results. Cystatin C is a novel agent that offers a sure promise as it is least influenced by factors that affect serum creatinine to a major extent. Aminoglycoside clearance, although still in the dark area, may prove a simple yet precise way of estimating glomerular filtration rate in those patients in whom these drugs are therapeutically employed. Optic ratiometric method has emerged as the most sophisticated one in glomerular filtration rate estimation in critically ill patients.

## Introduction

Critically ill patients with acute kidney injury (AKI) do differ from patients who are not ill in so many ways. Many mathematical formulae do exist to estimate the glomerular filtration rate (GFR) in this population of patients. AKI is common among hospitalized patients, especially in critically ill ones [1–6]. A decline in kidney function contributes to the accumulation of many drugs [7–9]. An accurate assessment of kidney function is required to optimize drug administration as both overdosing and underdosing of drugs may prove detrimental in a critically ill patient.

## Review

### Problems in GFR estimation in an intensive care unit (ICU) set up

Critically ill patients often have rapidly fluctuating renal hemodynamics in the setting of AKI, and changes of GFR are poorly reflected by daily changes in serum creatinine concentrations in patients with AKI [10]. Creatinine as a single parameter always has demerits in non-steady states observed in ICU patients as it is always constantly released into the circulation, affected by many factors including muscle mass and drugs [11–14]. Moreover, creatinine poorly differentiates between pre-renal and intrinsic renal failures and approximately 50% of renal mass has to be lost for the serum creatinine to rise [15].

Often, the phenomenon of augmented renal clearance (ARC) in critically ill patients is either missed or not understood. This is seen in ill patients who are on vasopressors and deliberate fluid support. This is important in calculating the dosage of drugs especially those with narrow therapeutic range.

### ARC in critically ill: an enigmatic concept

ARC is defined as estimated GFR > 130 ml/min. In a study by Andrew Udy et al. [16], 17 of 20 patients (85%) with traumatic brain injury who required saline infusion and vasopressor agents to maintain the cerebral perfusion pressure had ARC. Using timed urinary creatinine clearance method to measure GFR, ARC was demonstrated in burns [17, 18], sepsis [19] and surgery, and in ICU patients. Brown et al. [20] recorded a creatinine clearance of 190 ml/min/1.73 m^2^ in a cohort of critically ill postoperative patients. Recently, Fuster-Lluch et al. [21] reported an incidence of 17.9% in a cohort of postoperative and polytrauma patients, who were younger, with lower Acute Physiology And Chronic Health Evaluation (APACHE) II scores, higher diastolic blood pressures, and higher urine output on the first morning of admission to the ICU. Cytokine release from acute injury [22], the innate immune and inflammatory responses to trauma [23], and aggressive fluid resuscitation may promote increased organ blood flow and enhanced excretory function. The importance of the concept is quite obvious. This throws light on the doses of renally excreted drugs and may mean that standard dosing regimens are inadequate in patients with ARC. Baptista et al. [24] examined the performance of four creatinine-based estimation equations in 86 patients with ARC. This *post hoc* analysis showed that all the better known GFR equations underestimated the measured creatinine clearance value. The list below illustrates the practical problems in estimating GFR in an ICU setup:

1. Rapidly fluctuating renal hemodynamics
2. Both overdosing and underdosing are problems when GFR could not be estimated close to accuracy
3. Constantly changing volume status of the patient
4. Concept of ARC is not frequently applied in calculating GFR leading to inadequate dosing of drugs
5. Commonly used creatinine-based equations are flawed in the critical care setting. Ideal methods like inulin clearance become impractical in an ICU setup

### Equations for estimating creatinine clearance/GFR

#### CG formula

(140 − Age in years) × Body weight in kg/72 × Serum creatinine in mg/dl. If the patient is a female, multiply result by 0.85 (Table one in [25]).

#### The 4-variable MDRD



If a patient is female, multiply result by 0.742 and if black, multiply result by 1.212 [25].

#### The 6-variable MDRD



If female, multiply result by 0.762 and if black, multiply result by 1.18 [25], where BUN = blood urea nitrogen measured in mg/dl, Alb = serum albumin concentration measured in g/dl.

Only few studies have attempted to improve estimation of GFR in critically ill patients and these studies included subjects with stable kidney function in various settings [17, 26–28].

Relatively accurate estimation of GFR in a non-steady state as in AKI requires timed urine collections, which is not always practically possible in a critically ill patient. To overcome this, Jelliffe introduced an equation in 2002 [29, 30].

#### Jelliffe's equation

Estimated GFR = {(Volume of distribution × (Serum creatinine on day 1 − Serum creatinine on day 2)} + Creatinine production) 100/1,440/Average serum creatinine [29, 30]*.*

Creatinine production (mg/day) is computed using the following equation:

[29].

Jelliffe's equation obviously takes into account the fluctuations of serum creatinine over time, which frequently occurs in AKI in ICU and which is not considered in the above-mentioned Cockcroft-Gault (CG) and Modification of Diet in Renal Diseases (MDRD) formulae. Ongoing creatinine production is also taken into consideration, a major merit. However, this does not consider the fluid balance variations that could also affect the serum creatinine [31]. Creatinine is a water-soluble substance, and rapid, especially overzealous fluid administration can lower the creatinine and falsely overestimate the GFR [32]. To overcome this, a modification was made, which adjusted each measured creatinine to cumulative fluid balance. This requires the employment of an entity called correction factor. This was referred to as modified Jelliffe's equation [29].

#### Modified Jelliffe's equation



[29].

The 24-h creatinine clearance is usually determined from a 24-h urine collection [32].

#### An example

In a 60-kg woman, if the serum creatinine is 1.4 mg/dl, urine creatinine is 110 mg/dl, and urine volume is 1.4 l/day, then creatinine clearance = [110 × 1.4]/1.4 = 110 l/day. This value has to be multiplied by 1,000 to convert into milliliter and then divided by 1,440 (the number of minutes in a day) to convert into units of milliliter per minute. Thus, creatinine clearance = [110 × 1,000]/1,440 = 76.4 ml/min.

In a study known as PICARD (Programme to Improve Care in Acute Renal Dysfunction) by Josee Bouchard et al. [33], the GFR was estimated by CG, MDRD, Jelliffe's, and modified Jelliffe's equations and was compared with measured urinary creatinine clearance. Twelve non-dialyzed, non-oliguric patients with consecutive increases in creatinine for at least 3 and up to 7 days were identified and taken into study. The GFR estimated by Jelliffe's and MDRD equations correlated best with urinary creatinine clearance. The degree of overestimation of GFR by CG, MDRD, and Jelliffe was 80%, 33%, and 10%, respectively, and the degree of underestimation of GFR of modified Jelliffe's equation was 2%. The relative overestimation of GFR in AKI with both CG and MDRD will be more prominent when baseline GFR is higher. Small absolute changes in serum creatinine will be reflected as large relative changes in GFR with a lower serum creatinine. Patients with fluid accumulation had GFR overestimated the most by CG and MDRD equations. In practice, it is imperative to adjust GFR for creatinine production and fluid balance at the beginning of an AKI episode, when the initial serum creatinine is within the normal or in the near-normal range.

Jelliffe's and modified Jelliffe's equations can be easily integrated in a computer program to facilitate dosage regimens of drugs that have a narrow therapeutic index. It is important to highlight the magnitude of variation among these available methods because GFR estimation in AKI could be used to adjust drug dosing and could also influence the timing of initiation of renal replacement therapy.

### Cystatin C - a real boon in GFR estimation

Cystatin C is a non-glycosylated protein produced by all nucleated cells at a constant rate [34]. Its constant rate of production [35], low molecular weight of 13 kDa, and positive charge at physiological pH makes it a suitable marker for glomerular filtration. It is reabsorbed and almost completely catabolized in the proximal tubule [34, 35]. It is found in relatively high concentrations in many body fluids, especially in the seminal fluid, cerebrospinal fluid, and synovial fluid [36, 37]. It has multitude of advantages including its extreme sensitivity to small changes in GFR and higher diagnostic accuracy than MDRD or CG formulae. Moreover, its concentration is least affected by infections, malignancies, steroid therapy, inflammatory disorders, and muscle mass [38–45]. In a study by Patricia Villa et al. [46], serum creatinine, serum cystatin C, and 24-h creatinine clearance were determined in 50 critically ill patients (age 21–86 years; mean APACHE II score 20 ± 9). Data showed that serum cystatin C correlated better with GFR than creatinine (1/cystatin C versus creatinine clearance, *r* = 0.832, *P* < 0.001; 1/creatinine versus creatinine clearance, *r* = 0.426, *P* = 0.002). Cystatin C was diagnostically superior to creatinine (area under the curve for cystatin C 0.927 and for creatinine 0.694). Half of the patients had AKI. Only 5 (20%) of these 25 patients had elevated serum creatinine, whereas 76% had elevated serum cystatin C levels (*P* = 0.032). This suggests that serum cystatin C is a better marker of GFR than serum creatinine in critically ill patients.

Two equations can be used to estimate GFR from cystatin C concentration:

(A) Grubb's equation [47]: GFR = 83.93 × Cystatin C^−1.676^
(B) Larsson's equation [48]: GFR = 77.239 × Cystatin C^−1.2623^; cystatin C is measured in mg/l.

### CG and 4-variable MDRD: woefully inadequate in the critical care setting

The CG formula was derived from creatinine clearance based on 24-h urine collections in 249 subjects in a medical ward in a veterans administration facility, for which 96% of patients were men mostly with mild renal dysfunction. The MDRD equation has its origin from a cohort of outpatients based on ^125^I iothalamate clearances with moderate-to-severe renal impairment. An abbreviated 4-variable MDRD equation uses only age, sex, race, and serum creatinine level to estimate the renal function. None of these equations has been thoroughly validated among ill hospitalized patients [49, 50]. The 6-variable MDRD equation also adds albumin and BUN concentrations into the model. This may be a more appropriate equation in a critical care setup. In a study by Poggio et al. [10], iodine-125 iothalamate clearances performed in 107 sick in-patients with renal dysfunction were compared with estimated GFR from the 6- and 4-variable MDRD and CG equations. Patients were also subdivided into those having BUN/creatinine ratio of >20 and <20. Compared with iothalamate GFR, the CG and MDRD formulae performed poorly with respect to their ability to predict actual GFR, as shown by their poor agreement and high degree of bias. The 6-variable MDRD equation showed a better concordance correlation coefficient compared with the 4-variable MDRD equation and CG formula (0.66 versus 0.57 and 0.46, respectively; *P <* 0.01). High BUN/creatinine ratios are the usual findings in hospitalized patients, and estimation of GFR in this population does pose a great challenge for the clinicians. In such situations, the 6-variable MDRD may serve as a good option than the other mathematical formulae in arriving at a relatively accurate GFR whereas the same may not offer any advantage over 4-variable MDRD in healthier subjects with preserved BUN/serum creatinine ratio [51–60].

### Chronic kidney disease-epidemiology (CKD-EPI) equation in critically ill patients

CKD-EPI collaboration developed a new equation to measure GFR based on serum creatinine, age, gender, and race:

[61] where *k* is 0.7 for females and 0.9 for male patients, *a* is −0.329 for female patients and −0.411 for male patients, min indicates the minimum of creatinine/*k* or 1, and max indicates the maximum of creatinine/*k* or 1). In comparison to the MDRD equation, CKD-EPI has greater precision and reliability, especially for GFR >60 ml/min per 1.73 m^2^. However, to date, this equation has not been evaluated in hospitalized patients. As its outcome is based on serum creatinine, overestimation of GFR does occur with CKD-EPI equation too, especially in cirrhotic patients with low muscle mass, although much less when compared with CG formula.

### Aminoglycoside clearance in urine as a measure of GFR in critical care patients: where do we stand?

Aminoglycosides might be used to estimate renal function because they are freely filtered, neither secreted nor reabsorbed in the kidney, and have little non-renal clearance. To date, conflicting results have been produced by studies on ICU patients in estimating renal function, both good and poor. This method can be used to estimate renal functions in ICU patients who are therapeutically prescribed aminoglycosides, usually amikacin, gentamycin, and tobramycin according to the dosing standards. In a study by TE Jones et al. in an ICU [62], gentamycin and tobramycin clearance was compared with standard MDRD and CG formulae along with serum cystatin C. These aminoglycosides were administered over 30 min using a computerized pump, and the first blood sample taken 30 min later. The second sample was taken at a time estimated to be twice the half-life of the drugs. These blood samples were also used in the calculations of creatinine clearance from the timed urine collections. The 2-h urine collection was commenced when the aminoglycoside infusion started, and the 24-h collection commenced at the end of the 2-h collection. Gentamycin clearance performed the best, being within 10% of the value on 44% of occasions and within 20% on 78% of occasions. Gentamycin clearance has previously been shown to be a better estimate of creatinine clearance than the 24-h urine estimate in a study using inulin as the comparator [63–66]. Giving aminoglycosides for the estimation of renal function alone may not be a wise option but in patients who are therapeutically prescribed aminoglycosides, their clearance may serve as one of the better options in estimating renal function.

### Optic ratiometric method for GFR estimation: do the advantages outweigh the question of its feasibility in an intensive care setup?

Novel methods for the rapid estimation of GFR include employment of portable fiberoptic ratiometric fluorescent analyzer, predominantly tested in large animal models, mainly in pigs and dogs, the two species in which the results are supposed to approximate in humans. In this novel method tested in animal models, an optical fiber of <1-mm size that delivers excitation light and absorbs fluorescent emissions is inserted into a central vein through a commercial intravenous catheter. A mixture of fluorescent chimeras of a small freely filterable reporter and large non-filterable plasma volume marker is infused as a bolus, excited by light-emitting diodes, and the *in vivo* signals were detected and quantified by photomultiplier tubes in the tested species within an hour. While the fluorescent chimera is used as a plasma volume marker, the small highly fluorescent and highly filterable reporter molecule is used to quantify the rate of its plasma clearance, which in turn is the measure of GFR. This method allows for the rapid, inexpensive, safe, reproducible, and patient-friendly method of estimating GFR in conditions where only a short time frame is available to make clinical decisions, as in AKI in a critical care setting. Exing Wang et al. [67], in their animal model study, found that the portable fiberoptic ratiometric fluorescent analyzer provided a rapid point-of-care determination of GFR which was standardized against the 6-h iohexol clearance from the plasma. Table 1 summarizes the overall features and characteristics of the parameters and equations that could be employed to estimate GFR in an ICU setup.

**Table 1:** **Salient features of various methods that could be employed to measure GFR in ICU setup**

Sl no	Methods	Merits	Demerits
1	CG formula	Easily computable	Highly inaccurate in the critical care setup. Considerable degree of GFR overestimation
2	4-variable MDRD	More accurate than CG. May offer value close to 6-variable MDRD in healthier patients with preserved BUN/Cr ratio	Dependency on creatinine. May not be accurate when BUN/Cr ratio is increased. Does not take into account blood urea nitrogen and albumin. Overestimation of GFR when baseline GFR is high
3	6-variable MDRD	BUN and serum albumin are taken into account. More accurate when BUN/CR ratio is increased. Better concordance correlation coefficient when compared with CG and 4-variable MDRD	Dependency on creatinine. Ongoing creatinine production and its fluid balance variations are not taken into account. Less accurate when compared with cystatin C and novel methods
4	CKD-EPI formula	Greater precision and reliability when compared with MDRD. More accurate when GFR >60 ml/min/1.73 m^2^	Not validated extensively in hospitalized and sick individuals. Dependency on serum creatinine
5	24-h creatinine clearance	More accurate when compared to CG and MDRD formulae	Collection of urine is an issue. Cannot provide immediate results. Becomes a problem when rapid administration of drugs is essential
6	Jelliffe's equation	Ongoing creatinine production and fluctuations in creatinine concentration over time are taken into account	Does not take into account the variations in creatinine concentration with respect to fluid balance
7	Modified Jelliffe's equation	Fluid balance variations of creatinine are also taken into account	Still less accurate when compared with cystatin C and fiberoptic radiometric methods
8	Serum cystatin C	Less affected by non-renal factors. Sensitive to changes in so-called creatinine blind GFR(40–70 ml/min); preferred agent in liver disease patients	Expensive and unreliable in the presence of thyroid dysfunction, may be affected in patients taking high dose steroids with renal dysfunction
9	Aminoglycoside clearance	May serve as an easy method for GFR estimation in patients already on aminoglycosides. May be better than 24-h creatinine clearance	Giving aminoglycoside for renal function estimation alone may not be wise or practically possible
10	Fiberoptic ratiometric fluorescent analyzer method	Most accurate way of all. Rapid, inexpensive, reproducible, and safe method	Still experimental. Requirement of technical expertise. Scarce studies in humans

## Conclusions

The concept of estimating renal function in critically ill patients had always remained elusive. The issue of ARC should be considered to avoid improper dosing of drugs. CG and 4-variable MDRD equations should better be avoided in ICU setup. Cystatin C, modified Jelliffe's, Jelliffe's, and 6-variable MDRD equations could be employed in that order to estimate renal function and to provide instant and accurate results. While aminoglycoside clearance could be used in patients in whom they are therapeutically used, optic ratiometric method provides the most accurate and instant estimation of renal function in critically ill patients, although large-scale studies in humans are required to validate it.

## Abbreviations

AKI: acute kidney injury
APACHE: acute physiology and chronic health evaluation score
ARC: augmented renal clearance
BUN: blood urea nitrogen
CG: Cockcroft-Gault
CKD-EPI: chronic kidney disease-epidemiological equation
GFR: glomerular filtration rate
ICU: intensive care unit
MDRD: modification of diet in renal diseases.
